# Soziale Arbeit und Krise: Professionelle Bearbeitungsweisen und gesellschaftliche Thematisierungen

**DOI:** 10.1007/s12054-021-00401-y

**Published:** 2021-06-29

**Authors:** Martina Richter, Katharina Sufryd, Meike Wittfeld

**Affiliations:** Universität Duisburg-Essen, Essen, Deutschland

**Keywords:** Krise, Soziale Arbeit, Krise als Narrativ, Kinderschutz, Gemeinwesenarbeit, Jugendhilfe, Wohnungslosenhilfe, Corona-Pandemie

## Abstract

Die Bezeichnung eines gesellschaftlichen Phänomens als Krise ist wirkmächtig. Durch die Klassifizierung wird ein Handlungsdruck markiert, der sowohl produktiv als auch hinderlich sein kann. Die Soziale Arbeit als Disziplin und Profession ist Teil der gesellschaftlichen Verhältnisse, in denen Krisen entstehen und bearbeitet werden. Im vorliegenden Schwerpunkt wird diese Eingebundenheit theoretisch-systematisierend entfaltet und zudem für drei ausgesuchte Arbeitsfelder der Sozialen Arbeit reflektiert.

Die Klassifizierung von gesellschaftlichen Phänomenen als Krise ist allgegenwärtig. Doch was genau ist eigentlich eine Krise? Und in welchem Verhältnis steht die Soziale Arbeit zu Krisen?

Die Thematisierung gesellschaftlicher Ereignisse als Krise ist allgegenwärtig. Im Zuge der Verbreitung des Coronavirus SARS-Cov‑2 ist öffentlich von einer ‚Corona-Krise‘ die Rede. Aber auch weitere gesellschaftliche Ereignisse werden als Krisen ausgewiesen. So findet mindestens seit 2007/08 der Begriff der ‚Finanzkrise‘ Eingang in öffentliche Debatten und seit 2015/2016 wird eine sogenannte ‚Migrationskrise‘ verhandelt. Die Aufzählung gesellschaftlicher Ereignisse, die zur Krise erklärt werden, ließe sich fortsetzen. Krisen scheinen immer wieder auf ein Neues ausgerufen zu werden. Auf den ersten Blick könnte der Eindruck entstehen, mit Krise ließe sich dem Verständnis nach etwas selbstverständlich Gegebenes benennen. Bei näherer Betrachtung handelt es sich jedoch um sehr unterschiedliche gesamtgesellschaftliche Ereignisse, die mit dem Begriff der Krise umrissen werden. Insofern ist vielmehr davon auszugehen, dass es sich bei einer Krise um ein Phänomen handelt, das immer historisch spezifisch hervorgebracht wird und in einen jeweiligen gesellschaftlichen Kontext zu stellen ist. Dabei ist die Bezeichnung eines Phänomens als Krise ein machtvoller Vorgang. Denn durch die Benennung als Krise kommt einem Phänomen oder einem Ereignis zumeist eine hohe öffentliche Aufmerksamkeit mit alarmierender Wirkung zu, verbunden mit einer Aufforderung zum unmittelbaren Handeln (vgl. Bösch et al. 2020; Dollinger 2006, 2013; Graf 2020).

In der Zuschreibung, was eine Krise ist und was nicht, zeigt sich demnach in zugespitzter Weise, wie Ereignissen und Zuständen eine unterschiedliche gesamtgesellschaftliche Relevanz zukommt. Dies hat zum Teil weitreichende Konsequenzen. So scheint etwa im Zuge der ‚Klima-Krise‘ noch darum gekämpft werden zu müssen, auch als solche wahrgenommen zu werden und durch diese Klassifizierung Akteur_innen zum Handeln zu bewegen (vgl. Katsandiou 2021).

Soziale Arbeit ist in gesellschaftliche Krisendynamiken eingebunden und wird gerade auch als Instanz der Krisenbearbeitung adressiert. Wie diese Adressierung in verschiedenen Arbeitsfeldern Sozialer Arbeit zum Ausdruck kommt und welche Folgen sich für Adressat_innen und Nutzer_innen, aber auch für eine professionelle Praxis zeigen, wird in den folgenden Beiträgen diskutiert und kritisch reflektiert. In diesem Schwerpunk wird insofern danach gefragt werden, was unter welchen gesellschaftlichen Bedingungen zur Krise erklärt wird und zu welchen gesellschaftlichen Verwerfungen und Ausschlüssen die Markierung von Ereignissen als Krise führen kann. Weiterhin soll diskutiert werden, welche Dynamiken sich mit Blick auf Krisen in den Feldern der Sozialen Arbeit zeigen, welche Wirkmächtigkeit können sie entfalten und welche Funktion kommt ihnen zu.

Dabei dient der erste Beitrag von Bernd Dollinger einer theorie-systematischen Erkundung von Krise und ihrem Erscheinen in der Sozialen Arbeit. Dollinger rückt vor allem die Thematisierung von gesellschaftlichen Krisenzuständen in der Sozialpädagogik in den Vordergrund und setzt sich mit deren Konsequenzen auseinander. Er stellt heraus, dass Krisendiagnosen Teil dominanter Narrative sind, die nicht selten mit der Aufforderung zur Integration einzelner (Personen‑) Gruppen in die vermeintlich beständige Gemeinschaft einhergeht. Die Nebenfolgen einer derartigen durch die Sozialpädagogik unterstützten Krisenbeschreibung werden exemplarisch u. a. veranschaulicht an der Gegenwartsdiagnose einer so genannten ‚Migrationskrise‘. An diesem diskursiven Phänomen wird aufgezeigt, dass die Markierung von (Personen‑) Gruppen u. a. zur Kriminalisierung von Menschen mit Fluchterfahrungen führt. Dollinger verweist damit auf ein dominantes Narrativ, das sich in die strukturellen Bedingungen von Rassismus eingebettet sehen lässt.

Die drei weiteren Beiträge von Ella und Noelle O’Brien-Coker (DEMASK), Nina Kläsener und Manuela Grötschel nehmen jeweils ein spezifisches Feld der Sozialen Arbeit in den Blick, an dem exemplarisch aufgezeigt wird, welche Umgangsweisen mit Blick auf gesellschaftliche Krisen sichtbar werden und vor welche Herausforderungen sich die pädagogischen Fachkräfte und die Institutionen durch diese Krisen gestellt sehen.

Vor dem Hintergrund der eigenen Praxis rücken Ella und Noelle O’Brien-Coker das grundlegende Dilemma von ehrenamtlicher Gemeinwesenarbeit in den Fokus, wonach zivilgesellschaftliche Arbeit nicht zu leisten wäre, ohne dominanten Diskursen zu folgen und damit zugleich eine zuschreibende und pauschalisierende Kategorisierung von (Personen‑) Gruppen und deren Marginalisierung zu affirmieren. Sie rücken in ihrem Beitrag die Schwierigkeit der Anerkennung und die prekäre Institutionalisierung in der Organisation von zivilgesellschaftlicher Arbeit unter den strukturellen Bedingungen von Rassismus, Heteronormativität sowie Queer- und Trans*-Feindlichkeit in den Vordergrund. Als Bearbeitungsstrategie wird eine dekonstruktive Perspektive vorgeschlagen, die die Komplexität gesellschaftlicher Bewegungen und Missstände sichtbar machen will.

Der Beitrag von Nina Kläsener regt zur Reflexion sozialpädagogischer Interventionen im Feld der Kinder- und Jugendhilfe an. Ausgehend von der Beobachtung, dass eine Fokussierung in der Kinder- und Jugendhilfe zunehmend auf dem Kinderschutz liege, werden in dem Artikel die Entscheidungsdilemmata in der sozialpädagogischen Praxis und die Unmöglichkeit einer eindeutigen Bestimmung des Kindeswohls herausgearbeitet.

Insbesondere durch eine Praxis der Inobhutnahme zeige sich eine ausgeprägte Dramatik, hervorgebracht durch Schutzlogiken, die auf einer Konstruktion von „Kindeswohlgefährdung“ als Krise beruhen.

Die Frage des Schutzes von Jugendlichen innerhalb bestehender sozialpädagogischer Angebotsstrukturen steht auch im Fokus des Beitrags von Manuela Grötschel. Dabei reflektiert sie die Bedingungen angesichts der gegenwärtigen „Corona-Krise“. Am Beispiel eigener Beobachtungen im sozialpädagogischen Feld der Jugend- und Wohnungshilfe veranschaulicht Grötschel ein Scheitern einer allein an Adressat_innengruppen ausgerichteten Bearbeitung von Problemlagen. Das Ausmaß einer gesamtgesellschaftlichen Krise im Zuge der Corona-Pandemie werde im Feld der Jugend- und Wohnungslosenhilfe besonders sichtbar, da die Inanspruchnahme der Angebote Rückschlüsse auf die Notlage der Jugendlichen unmittelbar zulasse. Insbesondere in diesem Handlungsfeld werde deutlich, dass die prekären Lebenslagen von jungen Menschen auf einer strukturellen Ebene unbearbeitet gelassen werden, sich in der Krise weiter zuspitzen und darüber veränderte Anforderungen an die Professionalität in dem Feld stelle, die fachlich und politisch beantwortet werden müssen.

Die vier Beiträge geben auf unterschiedliche Art Einblicke in die Klassifizierung von gesellschaftlichen Phänomenen als Krise. Die Eindrücke aus den unterschiedlichen Handlungsfeldern machen deutlich, dass die Soziale Arbeit aufgrund ihrer Position und auch Expertise als Krisenarbeiter_in adressiert wird. Gleichzeitig steht die Soziale Arbeit durch die Bearbeitung von Krisen auch in der Gefahr, sich an der (Re‑) Produktion einer eben auch problematischen Krisenartikulation zu beteiligen, die es kritisch zu reflektieren gilt.
